# The role of lifestyle factors in the association between education and self-reported fibromyalgia: a mediation analysis

**DOI:** 10.1186/s12905-024-03060-9

**Published:** 2024-04-17

**Authors:** Faith Owunari Benebo, Marko Lukic, Monika Dybdahl Jakobsen, Tonje Bjørndal Braaten

**Affiliations:** 1 Department of Community Medicine, UiT The Artic University of Norway, Tromsø, Norway; 2 Department of Health and Care Sciences, UiT The Arctic University of Norway, Tromsø, Norway

**Keywords:** Fibromyalgia, Lifestyle factors, Mediation analyses, Body mass index, Physical activity, Smoking, Alcohol consumption, Socioeconomic position, Education

## Abstract

**Background:**

Socioeconomic status as measured by education, income, or occupation, has been associated with fibromyalgia but the underlying mechanism and the role of lifestyle factors are unclear. Thus, we examine the role of modifiable lifestyle factors (body mass index, physical activity, alcohol consumption and smoking) in the association between education and self-reported fibromyalgia.

**Methods:**

We used data from 74,157 participants in the population-based prospective Norwegian Women and Cancer (NOWAC) study. Socioeconomic position, operationalized as years of educational attainment, and lifestyle factors were assessed via self-reported questionnaires. Multiple mediation analysis was used to decompose total effects into direct and indirect effects. Estimates were reported as hazard ratios (HRs) with 95% confidence intervals (CIs).

**Results:**

The cumulative incidence of fibromyalgia was 3.2% after a median follow up time of 13 years. Fibromyalgia was inversely associated with years of educational attainment for ≤ 9 years (HR = 2.56; 95% CI 2.32–2.91) and for 10–12 years (HR = 1.84; 95% CI 1.72–2.02), compared with ≥ 13 years of education. Overall, all lifestyle factors together jointly mediated 17.3% (95% CI 14.3–21.6) and 14.1% (95% CI 11.3–18.9) of the total effect for ≤ 9 years and 10–12 years of education, respectively. Smoking and alcohol consumption contributed the most to the proportion mediated, for ≤ 9 years (5.0% and 7.0%) and 10–12 years (5.6% and 4.5%) of education.

**Conclusion:**

The association between education and self-reported fibromyalgia was partly explained through lifestyle factors, mainly smoking and alcohol consumption.

## Introduction

Fibromyalgia syndrome (FM) is a common chronic condition characterized by chronic widespread pain, fatigue, and sleep disturbance; sufferers may also experience other functional and somatic symptoms [1]. Research has shown that low socioeconomic status is associated with FM [2, 3]. This is in line with similar patterns observed for other health conditions including chronic musculoskeletal conditions [4–6]. The association between social factors and health indicators/outcomes is well established and widely reported in the literature across various population groups [7–9]. Often, the observed association follows a gradient pattern; poorer health is observed among those with lower socioeconomic position, and vice versa [7, 8].

The socioeconomic gradient observed in many health outcomes have been attributed to material circumstances, psychosocial, behavioral, and biological factors. These factors act as intermediary determinants in the pathway between socioeconomic position (SEP) and health [7, 10]. The pathway is a complex web of relationships that are often non-mutually exclusive, and it is challenging to adequately measure all possible factors simultaneously. Nevertheless, research can be applied to understand different portions of the pathway [10–12].

Given that FM is a condition of unknown etiology and far-reaching consequences for the individual and the society at large [3, 13, 14], there is need for research to better understand the condition and identify opportunities for intervention. However, there is paucity of research on the mediating role of lifestyle factors in the pathway between SEP and FM. A mediation approach can be applied to explore the mechanisms that underlie the relationship between SEP and FM. Socioeconomic position is usually measured by individual level indicators (e.g., education, occupation, income, or an index that combines different socioeconomic indicators), household or structural level indicators (e.g., area and neighborhood level measures) [15, 16]. Education has been shown to have a stronger association with health behaviors than with material circumstances [7, 17–19]. Also, research suggests that lifestyle factors are associated with chronic musculoskeletal pain conditions [20–23], and the distribution of these lifestyle factors are often socioeconomically patterned [24, 25]. Thus, in this study, we use years of education as the measure of SEP and examine lifestyle factors as mediators. In the present study, we applied a mediation approach to examine the role of modifiable lifestyle factors (body mass index, physical activity, alcohol consumption and smoking status) in the association between education and self-reported FM.

## Methods

### The Norwegian women and cancer study (NOWAC)

**Fig. 1:**
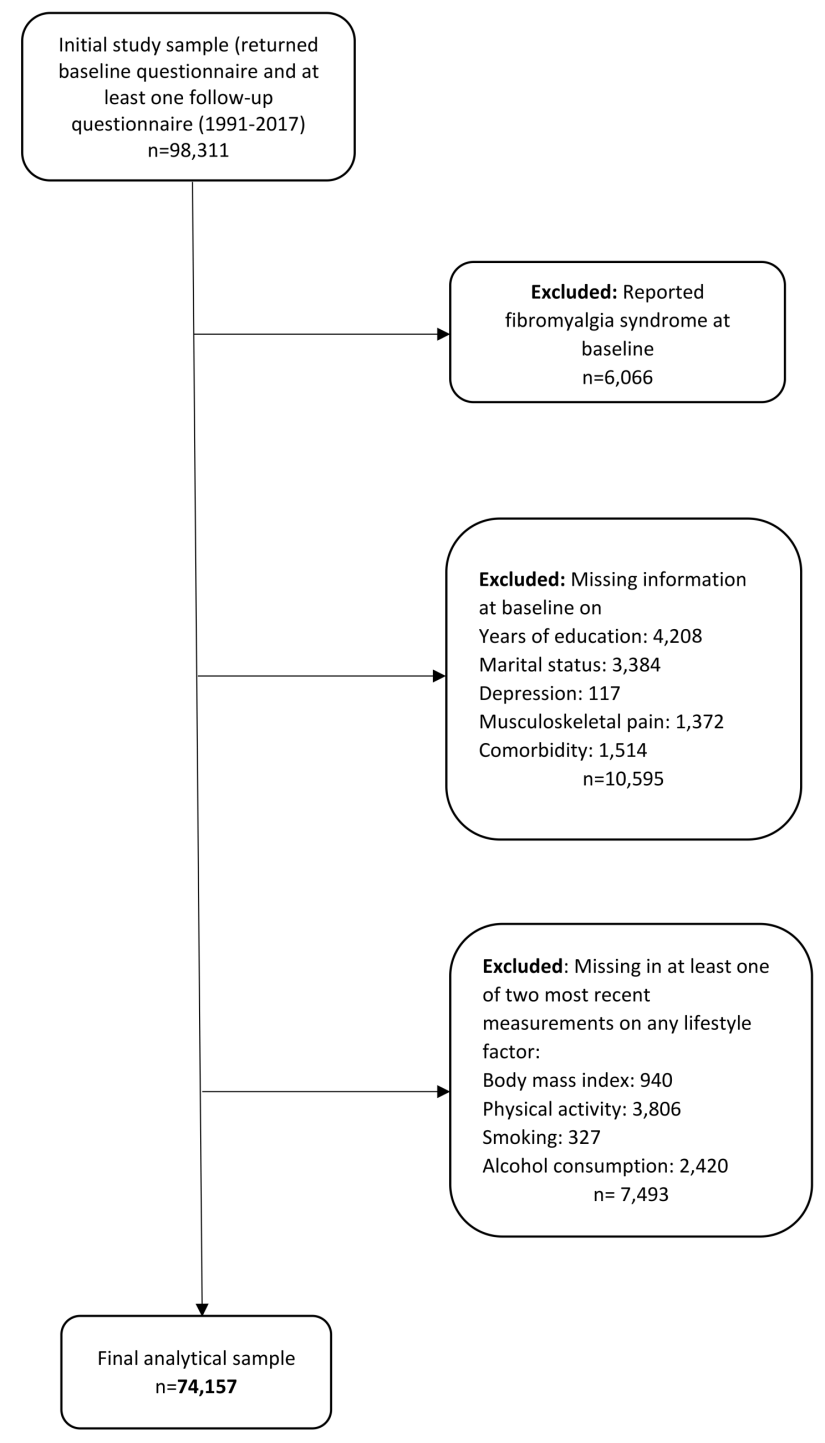
Flowchart of study participants

NOWAC is a nationally representative population-based cohort study consisting of about 172,000 women. The NOWAC study was established as a national population-based cohort to originally explore oral contraceptive use and other risk factors of breast cancer. However, the array of data collected has enabled the study of other cancer sites and health outcomes. Women aged 30–70 years were randomly sampled from the Norwegian Central Person Register and invited to participate in the study. Recruitment took place between 1991 and 97, with a response rate of 57%, and in 2003–06 with a response rate of 48.4%. Participants received follow-up questionnaires about every sixth to seventh year after enrollment. Details of the NOWAC cohort and validity studies are described elsewhere [26–29]. Data on years of education and covariates were collected at baseline, while data on lifestyle factors were collected at baseline and follow-up. The present study population is constituted by 98,311 women who have completed the baseline questionnaire (1991–1992, 1996–1997, or 2003–2004) and at least one follow-up questionnaire after enrollment. Women who reported FM at enrollment (*n* = 6,066) were excluded. We further excluded those with missing information on years of education (*n* = 4,208), marital status (*n* = 3,384), depression (*n* = 117), musculoskeletal pain (*n* = 1,372) and any comorbidity (*n* = 1,514) at baseline. We also dropped those with missing data in at least one of the two most recent measurements of lifestyle factors before self-reported FM or end of follow up, whichever came first; BMI (*n* = 940), physical activity level (*n* = 3,806), smoking status (*n* = 327), alcohol consumption (*n* = 2,420). Thus, the final analytical sample included 74,157 women (Fig. 1). We followed the AGReMA (A Guideline for Reporting Mediation Analyses) reporting guideline [30].

### Measures

The outcome variable, self-reported FM, was operationalized as the time from baseline until self-reported onset of FM or the last follow-up, whichever came first. At baseline, the question “Have you had any of the following illnesses: fibromyalgia … (among other conditions)?” was asked. Respondents who answered “yes”, further responded about the age at onset. At subsequent follow up waves, respondents were only instructed “For the following conditions: Fibromyalgia … (among other conditions), tick which year they emerged”.

The exposure of interest, years of education (referred to as education hereafter), and categorized based on the levels in the educational system in Norway as up to 9 years (primary school with at most two years of additional education), 10–12 years (may have completed secondary school, or up to five years of professional training), 13–16 years (university bachelor’s degree, or, in some instances, several professional training sessions at a lower level), more than 16 years (mainly corresponds to a university master’s degree level). Education was self-reported at baseline only.

Our proposed mediators (body mass index, physical activity, alcohol consumption and smoking) were self-reported at baseline and every follow-up; thus, participants had two or three measurements for each lifestyle factor. We used one of the two most recent measurements before self-reported FM or the end of follow-up, whichever came first. Body mass index (BMI) was calculated as weight in kilograms divided by height in meters squared and categorized as underweight (< 18.5 kg/m^2^), normal weight (BMI 18.5–24.9 kg/m^2^), overweight (BMI 25–29.9 kg/m^2^), and obese (BMI ≥ 30 kg/m^2^) [31–34]. Physical activity (PA) level was measured on a validated 10-point ordinal scale [28] and categorized as 1–2 very low; 3–4 low; 5–6 moderate; 7–8 high; 9–10 very high. Smoking status (stated as smoking hereafter) was categorized as never, former, and current. Alcohol consumption (stated as alcohol hereafter) in g/day was computed from reported intake of different beverages in predefined categories. The Norwegian Guidelines on Diet, Nutrition and Physical activity recommends 10 g/d as the daily limit for alcohol consumption for women [35]. Thus, alcohol was categorized as teetotaller; low (0.1–3.9 g/day); moderate (4.0–10 g/day); and high (> 10 g/day). Covariates in our models also included age (continuous), marital status (married/cohabiting vs. others), depression (yes vs. no), history of musculoskeletal pain (yes vs. no), menopausal status (pre- vs. post- menopause) and any comorbidity (yes vs. no).

### Statistical analyses

All statistical analyses were performed with STATA version 17.0 (Stata Corp, College Station, TX, USA) and using version 10 of the multiple mediation analysis (mma) package in R, version 4.2.2 (R Foundation for Statistical Computing). Descriptive statistics are presented as age adjusted means and standard deviations, while categorical data are presented as counts and age-adjusted proportions.

**Fig. 2:**
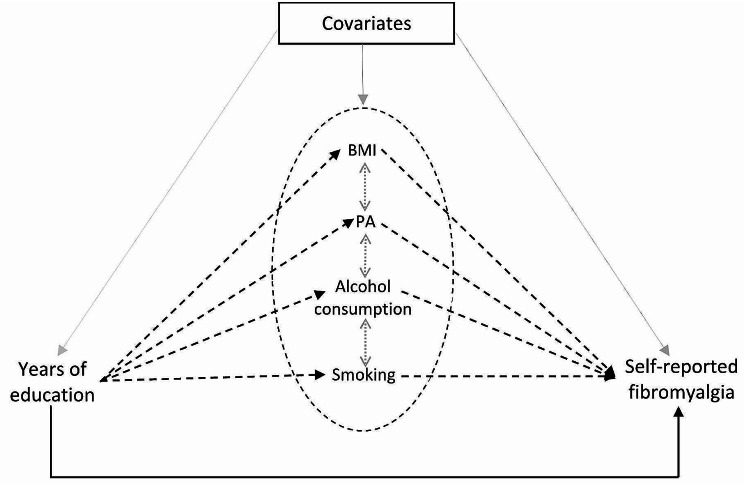
Conceptual model of the role of lifestyle factors in the relationship between years of education and self-reported fibromyalgia. BMI: Body Mass Index. PA: Physical Activity. Dashed lines (---): indirect effect; Solid line (–): direct effect. Bidirected dotted lines indicate that the mediators may be correlated. For clarity, all arrows between covariates and each lifestyle factors are not shown

Figure 2 shows the conceptual model of the assumed relationship among education, lifestyle factors (BMI, PA, smoking, and alcohol) and self-reported fibromyalgia. Our assumption is that educational attainment influences risky or health-promoting behaviours, which subsequently impact the risk of FM. Although the information of both education and covariates were collected at baseline, we consider education mainly to be completed at study enrollment and thus to precede the covariates and lifestyle factors measured in the study. We hypothesized that our chosen lifestyle mediators BMI, PA, smoking, and alcohol, are associated with education and self-reported FM [3, 36–39]

We conducted the mediation analyses, using the general multiple mediation analysis (mma) method for time-to-event outcomes [40, 41]. In this approach, we define our mediators (BMI, PA, smoking, alcohol) as intermediate variables that lie in the pathway between the exposure (education) and the outcome (self-reported FM), to provide insight into the mechanism through which the exposure influences the outcome. The total effect of the exposure (education) on the outcome (self-reported FM) can be decomposed into direct and indirect effects. The direct effect is defined as the effect of the exposure (education), on the outcome (self-reported FM), not through any of the mediators (BMI, PA, smoking, alcohol consumption); it can be interpreted as the remaining educational disparity in self-reported FM if the distribution of various lifestyle factors across educational groups could be equalized. The indirect effect is the effect of the exposure, on the outcome, through the mediators, individually or jointly. For a particular mediator, the indirect effect is the change in educational disparity, if the distribution of the mediator could be equalized between the reference group (≥ 13 years of education) and the other categories, while the distribution for the other mediators is kept as observed [40].

The definitions of the mediation effects are related to conventional mediation analysis but are more general as they are consistent for different types of predictors or outcomes [42]. With the adopted approach, multiple mediators can be considered simultaneously, yielding a joint indirect effect of all mediators together, and the indirect effects contributed by the different mediators individually. Thus, we can compare the relative effects of different mediators on the exposure. Also, with this approach, correlation among mediators is allowed, and we are not restricted by the assumption of ‘no interaction’ between exposure and mediators. This is important because lifestyle factors often cluster in individuals, thus they may be correlated or act individually [43, 44]. The bidirected dotted lines in the conceptual model (Fig. 2) indicate that the mediators may be correlated. Three assumptions are required for the general multiple mediation approach: no unmeasured confounder for the exposure-outcome relationship, no-unmeasured-confounder for the mediator-outcome relationship and no mediator is causally prior to other mediators [40, 42].

**Fig. 3:**
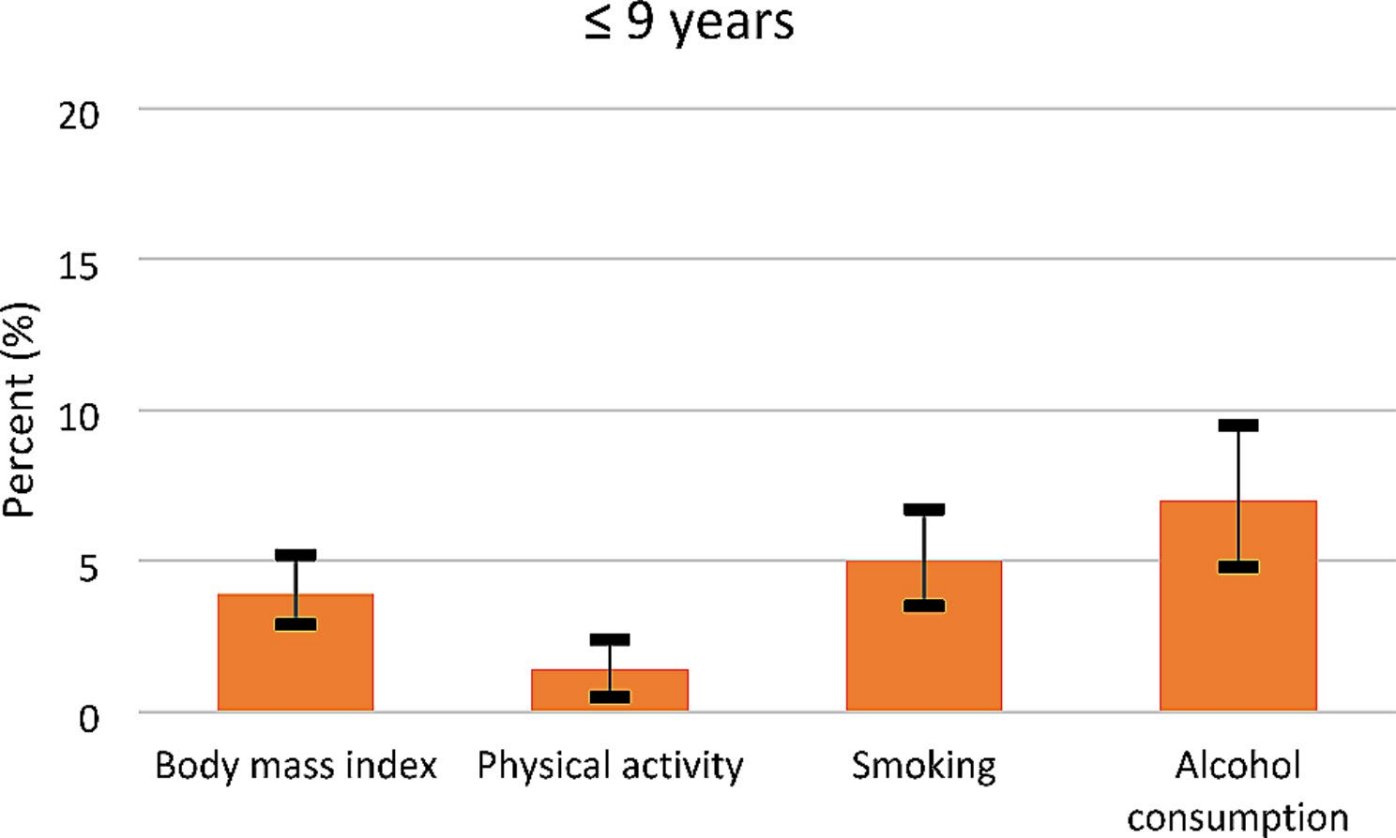
Proportion of the total effects mediated by lifestyle factors, in the association between education and self-reported FM, among women with ≤ 9 years of education

**Fig. 4:**
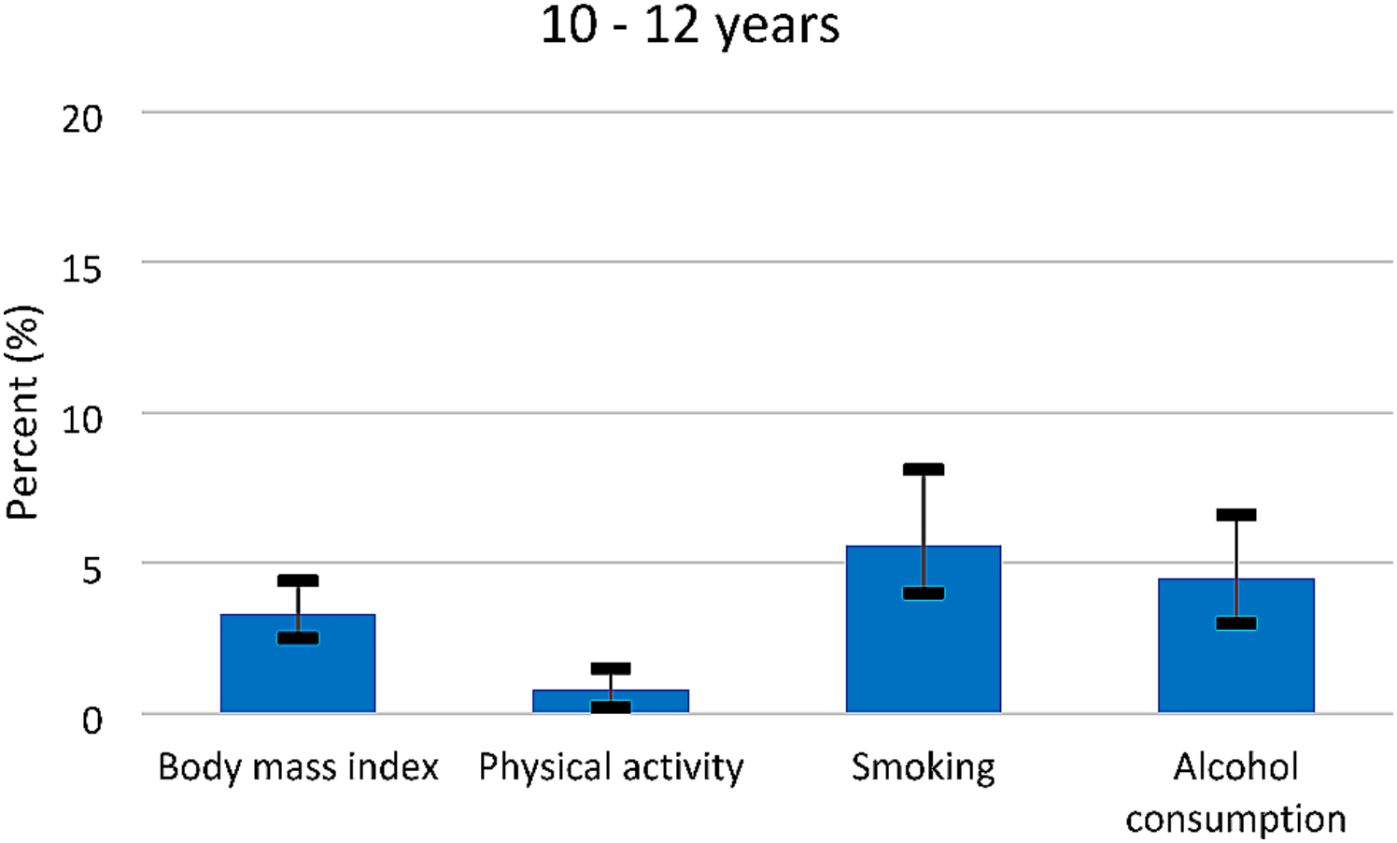
Proportion of the total effects mediated by lifestyle factors, in the association between education and self-reported FM, among women with 10–12 years of education

We describe baseline characteristics of the final analytical sample according to years of education (Table 1). Categorical variables are reported as frequencies with percentages, and continuous variables as means with standard deviations. Confidence intervals for the estimated mediation effects were derived using the bootstrap method. Given the time-to-event outcome, estimates of the mediation analyses are presented as hazard ratios (HRs) for the direct, indirect (joint and individual), and total effects with associated bootstrapped 95% CIs (Table 2). Mediation was indicated by the presence of a significant indirect effect and illustrated by the relative effects (Figs. 3 and 4). The relative effect was calculated by dividing the indirect effect by the total effect. The reference category for the mediation analyses was ≥ 13 years of education.

**Table 1:** Age-adjusted characteristics of the study sample (*N* = 73,433) by years of educational

Characteristics	All (*N* = 74,157) n (%)	Years of Education		
		≤ 9 (*n* = 15,599) n (%)	10–12 (*n* = 25,575) n (%)	≥ 13 (*n* = 32,983) n (%)
**Age at enrolment (years)** 30–39 40–49 50–59 ≥ 60	18,358 (24.8) 32,537 (43.9) 17,878 (24.1) 5,384 (7.3)	2,498 (16.0) 6,650 (42.6) 3,985 (25.6) 2,466 (15.8)	6,463 (25.3) 11,576 (45.3) 5,951 (23.3) 1,585 (6.2)	9,397 (28.5) 14,311 (43.4) 7,942 (24.1) 1,333 (4.0)
Mean age (± SD) in years at enrolment	46.4 (8.2)	49.1 (± 9.1)	46.0 (± 8.0)	45.3 (± 7.7)
**Body mass index (kg/m** ^**2**^ **)** Underweight (< 18.5) Normal weight (20.0-24.9) Overweight (25.0-29.9) Obese (≥ 30.0)	1,111 (1.5) 44,261 (60.0) 22,037 (29.7) 6,748 (9.1)	2,31 (1.6) 8,189 (53.7) 5,326 (32.9) 1,853 (11.6)	369 (1.4) 14,841 (57.9) 7,896 (30.9) 2,469 (9.7)	511 (1.5) 21,231 (64.0) 8,815 (27.0) 2,426 (7.4)
Body mass index (kg/m^2^), mean (± SD)	24.7 (± 3.9)	25.2 (± 3.2)	24.8 (± 4.1)	24.3 (± 4.6)
**Physical activity level** Very low (1–2) Low (3–4) Moderate (5–6) High (7–8) Very high (9–10)	2,830 (3.8) 14,833 (20.0) 31,160 (42.0) 20,996 (28.3) 4,338 (5.9)	901 (5.4) 3,252 (20.3) 6,289 (40.3) 3,959 (25.9) 1,198 (7.8)	963 (3.8) 5,095 (20.0) 11,060 (43.2) 6,989 (27.2) 1,468 (5.7)	966 (3.0) 6,486 (19.8) 13,811 (41.9) 10,048 (30.2) 1,672 (5.0)
**Smoking status** Never Former Current	26,782 (36.1) 27,608 (37.2) 19,767 (26.7)	4,660 (28.2) 5,354 (34.3) 5,585 (37.6)	8,090 (31.7) 9,516 (37.2) 7,969 (30.6)	14,032 (43.1) 12,738 (38.6) 6,213 (18.0)
**Alcohol consumption (g/day)** Teetotaler Low (0.1–3.9) Moderate (4.0–10) High (> 10)	5,878 (7.9) 42,249 (57.0) 18,342 (24.7) 7,688 (10.4)	1,897 (10.6) 10,350 (66.5) 2,530 (16.5) 822 (5.4)	1,793 (6.8) 15,291 (60.0) 6,182 (24.1) 2,309 (8.9)	2,188 (6.6) 16,608 (50.3) 9,630 (29.0) 4,557(13.6)
**Marital status** Married/cohabiting Others	62,618 (84.4) 11,539 (15.6)	13,252 (86.3) 2,347 (13.7)	21,993 (86.1) 3,582 (13.9)	27,373 (82.8) 5,610 (17.2)
**Depression** No Yes	62,442 (84.2) 11,715 (15.8)	13,045 (83.2) 2,554 (16.8)	21,609 (84.6) 3,966 (15.4)	27,788 (84.5) 5,195 (15.5)
**History of musculoskeletal pain** No Yes	63,959 (86.3) 10,198 (13.8)	12,867 (83.1) 2,732 (16.9)	21,898 (85.6) 3,677 (14.4)	29,194 (88.4) 3,789 (11.6)
**Any comorbidity** No Yes	55,954 (75.5) 18,203 (25.5)	10,772 (70.7) 4,827 (29.3)	19,165 (75.0) 6,410 (25.0)	26,017 (78.6) 6,966 (21.4)
**Menopausal status** Premenopausal Postmenopausal	38,386 (51.8) 35,771 (48.2)	6,787 (49.6) 8,812 (50.4)	13,362 (50.9) 12,213 (49.1)	18,237 (52.7) 14,746 (47.3)
**Self-reported Fibromyalgia** ^**1**^ No Yes	71,794 (96.8) 2,363 (3.2)	14,912 (95.5) 687 (4.5)	24,606 (96.8) 969 (3.2)	32,276 (98.3) 707(1.7)
**Event rate of self-reported FM (per 100,000 PY)**	235.1 (225.8–244.8)	370.4 (343.7–399.1)	278.2 (261.2–296.2)	150.0 (139.4–161.5)

^1^Outcome variable. SD: standard deviation. PY: person years. Proportions are age adjusted

We conducted sensitivity analyses to assess the robustness of our estimates to unmeasured confounding, using mediational E-values. The E-value measures how strong unmeasured confounders would be to substantially change the conclusions about the mediated effect [45]. Furthermore, to eliminate the influence of pre-existing disease on the estimated associations, we excluded those who reported FM within the first two years of the follow up. This was done because it may take an average of 2.3 years for symptomatic patients to receive a diagnosis of fibromyalgia [46]. We had missing data in years of education, lifestyle factors, and some covariates. Given that this is a prospective study, we assumed that missing data were not related to the outcome, and that complete-case analyses would suffice for the analyses. However, we still assessed the sensitivity of the findings from the primary analyses, when those with missing data were added to the sample, after multiple imputation. We conducted chained multiple imputation for missing data, then repeated the analyses based on 20 imputed datasets.

The Regional Committee for Medical and Health Research Ethics approved the present study. All research was performed in accordance with the ethical principles of the Declaration of Helsinki. Informed consent was obtained from all participants.

## Results

We observed a negative education gradient in BMI; overweight and obesity were least frequently observed among those with ≥ 13 years of education compared to those with 10–12 and ≤ 9years of education. Similarly, smoking showed a negative education gradient such that women with the most years of education reported more frequently to be never smokers or former smokers, while current smoking was observed most among those with ≤ 9years of education. The distribution of PA across levels of education showed similar trends for low and moderate levels of PA. However, we observed negative trends at the extremes of PA levels, and a positive education gradient for high level of PA. For alcohol consumption, there was a positive education gradient for moderate and high levels and a negative gradient for teetotallers and low level of alcohol consumption. Among those with ≤ 9 years of education, 4.5% of women reported FM, compared with 3.2% and 1.7% among those with 10–12 and ≥ 13 years of education, respectively.

During 1,005,169 person years of follow up, there were 2,363 cases of self-reported FM, and a median time to event of 6 (IQR = 5) years. The overall incidence rate was 235.1 per 100,000 person-years, with a cumulative incidence of 3.2%. The estimated direct, indirect, and total effects of education on the risk of self-reported FM, are shown in Table 2. The risk of self-reported FM was higher among women with ≤ 9 years of education (total effect, HR_TE_= 2.56; 95% CI 2.32–2.91 and women with 10–12 years of education (total effect, HR_TE_= 1.84; 95% CI 1.72–2.052), compared to their counterparts with ≥ 13 years of education. Among women with ≤ 9 years of education, the joint indirect effect of BMI, PA, smoking, and alcohol consumption was 17.3% (95% CI 14.3–21.6). This implies that 17.3% of the total effect was mediated by lifestyle factors (BMI, PA, smoking, and alcohol). The joint indirect effect (all lifestyle factors together) was 14.1% (95% CI 11.3–18.9), for women with 10–12 years of education, that is, all lifestyle factors together explained 14.1% of the total effect of years of education on the risk of self-reported FM.

**Table 2:** Mediating effects of lifestyle factors on the association between years of education and self-reported FM

Years of Education	HR (95% CI)	Relative effects (%)
**≤ 9 vs. ≥ 13** Total Effect (TE) Direct effect (DE) Indirect Effect (IE)^1^ IE: BMI IE: PA IE: Smoking IE: Alcohol	2.56 (2.32–2.91) 2.18 (1.98–2.47) 1.18 (1.14–1.21) 1.04 (1.03–1.05) 1.01 (1.01–1.02) 1.05 (1.03–1.06) 1.07 (1.04–1.08)	82.7 (78.4–85.7) 17.3 (14.3–21.6) 3.9 (2.9–5.2) 1.4 (0.5–2.4) 5.0 (3.5–6.7) 7.0 (4.8–9.5)
**10– 12 vs. ≥ 13** Total effect (TE) Direct effect (DE) Indirect Effect (IE)^1^ IE: BMI IE: PA IE: Smoking IE: Alcohol	1.84 (1.72–2.02) 1.69 (1.58–1.86) 1.09 (1.07–1.11) 1.02 (1.02–1.03) 1.01 (1.00–1.01) 1.03 (1.02–1.05) 1.03 (1.02–1.04)	85.9 (81.1–88.7) 14.1 (11.3–18.9) 3.3 (2.5–4.4) 0.8 (0.2–1.5) 5.6 (4.0–8.1) 4.5 (3.0–6.6)

HR: Hazard Ratio; CI: Confidence interval; BMI: Body mass index; PA: Physical activity ^1^Indirect effect of all mediators together (joint indirect effect). Mediation models are adjusted for age, marital status, depression, history of musculoskeletal pain, any comorbidity and menopausal status

For all categories of years of education, physical activity contributed the least, while smoking and alcohol consumption contributed the most in explaining the association between education and self-reported FM (Figs. 3 and 4).

Sensitivity analyses using E-values showed that to explain away the indirect effects of 1.18 among women with ≤ 9 years of education and 1.09 among those with 10–12 years of education, an unmeasured confounder associated with both years of education and self-reported FMS by hazard ratios of 1.64-fold (among women ≤ 9 years of education) and 1.40-fold (among women with 10–12 years of education) each, conditional on the measured covariates could suffice, but weaker confounding could not. This implies that a confounder, or set of confounders, would have to be associated with a 1.64-fold increase in the risk of self-reported FM, and must be 1.64 times more prevalent in those with ≤ 9years of education than those with ≥ 13years, to explain the observed hazard ratio. If the strength of one of these relationships were weaker, the other would have to be stronger for the effect of years of education on self-reported FM to be truly null. This also applies to the E-value of 1.40 for women 10–12 years of education.

To shift the lower CI to include the null value, an unmeasured confounder associated with years of education and self-reported FM by hazard ratios of 1.54-fold (among women ≤ 9 years of education) and 1.34-fold (among women with 10–12 years of education) could suffice, but weaker confounding could not.

Also in sensitivity analyses, the estimates did not change appreciably when we excluded participants (*n* = 220) who reported FM within the first two years of follow-up; thus, these participants were retained in the final analytical sample. For the missing data analyses, results from the imputed datasets and the complete-case analyses were similar, thus the results for the complete case analyses were presented.

## Discussion

The current study investigated the association between years of education and self-reported FM, and the mediatory role of lifestyle factors (BMI, PA, smoking, and alcohol consumption) in the observed association. Our results showed a negative educational gradient in self-reported FM, and all lifestyle factors together mediated part of the association between education and self-reported FM. The highest proportion mediated was attributable to alcohol for those with ≤ 9 years of education, while smoking mediated the highest proportion for those with 10–12 years of education.

There is paucity of comparable studies on the mediating role of lifestyle factors in the association between education and fibromyalgia. However, the literature shows that lifestyle factors may explain some of the socioeconomic disparities in musculoskeletal pain conditions and other health outcomes [6, 47–51]. Our findings align with studies that have reported positive associations between higher educational attainment and alcohol consumption [39]. Also, our results are consistent with studies that have shown an inverse association between alcohol consumption and reporting of chronic widespread pain, severity of fibromyalgia symptoms and quality of life quality of life [52–54]. This finding is the alcohol-harm paradox, which implies that those with lower SEP suffer more harm than those with higher SEP, even when they have similar or lower levels of alcohol consumption [55, 56]. This may be due to the presence of other health-damaging risk factors in those with lower SEP, which can interact with the exposure making them more vulnerable to poorer health outcomes than their higher SEP counterparts [44, 55–57].

We found that smoking, BMI, and PA also mediated the association between education and self-reported FM. The direction of the associations observed is consistent with findings from previous studies. Low level of education has been associated with increased uptake of smoking and decrease in smoking cessation [58, 59]. Smoking has also been associated with incident FM and other musculoskeletal pain conditions [60–62]. Similarly, lower levels of education have been associated with increased BMI [63], while normal BMI and physically active lifestyle have been linked to higher levels of education [38, 64]. Studies have also, reported significantly higher risk of FM and other musculoskeletal pain conditions among overweight and obese women [20, 65]. BMI and PA contributed the least in mediating the observed educational disparity. BMI is influenced by the interaction of genetic and other lifestyle factors including diet and physical activity [66]. Thus, it may not be the most appropriate measure of body fat or composition. On the other hand, studies have demonstrated non-significant and weak association between PA and FM [20, 67]. This may explain why the indirect effect of PA was almost non-existent.

The inverse relationship observed between education and self-reported FM is consistent with findings from previous studies [67, 68]. Similarly, low socioeconomic status has been associated with chronic widespread pain and other chronic musculoskeletal complaints [61, 69, 70]. Compared with their lower educated counterparts, people with higher levels of education tend to have better health literacy, and financial advantage with regards to adopting healthy behaviours [71, 72]. Together, BMI, PA, smoking, and alcohol consumption could not completely explain the association between education and self-reported FM. Other factors, for example psychosocial and work-related, may also be responsible for the educational disparity in self-reported FM. However, it is important to bear in mind that the larger socioeconomic and political context, and their structural mechanisms are the progenitors of individual socioeconomic positions. Thus, there is need to also explore multilevel and intersectoral approaches to interventions and policies targeted at reducing health inequalities [10, 73].

### Strengths and limitations

The strengths of our study include the use of nationally representative data and prospective nature of the study. As FM is a chronic condition which is likely to occur long after most people have completed their education, it is unlikely that there was health selection in the association between education and self-reported FM. Although, some women may have been experiencing symptoms of FM at baseline but not diagnosed, as it can take an average of 2.3 years after experiencing symptoms to receive a diagnosis [46], we accounted for this in the sensitivity analysis by excluding those who reported FM within the first two years of follow up. Lastly, the approach used for mediation analyses accommodated multiple mediators and allowed for decomposition of estimates, yielding the contribution of each lifestyle factor singly [40, 42].

However, some limitations should be kept in mind while interpreting the results, and causal interpretation should be avoided. Data on FM were obtained from self-report, in response to a single question. The question has not been formally validated, and there was no confirmatory diagnosis of FM in the study setting. Secondly, lifestyle factors were self-reported, as such, we cannot rule out measurement errors or reporting bias, which may have led to misclassification. However, validation studies have been conducted for the BMI and PA measures in the NOWAC study [28, 74]. Furthermore, epidemiological studies usually collect data on lifestyle factors via self-reports, as it is often the most feasible method. Also, we cannot completely rule out selection bias that may arise from non-response. This is because non-responders may be systematically different from those who responded [75]. Thirdly, we assumed that educational attainment preceded lifestyle and the subsequent occurrence of FM. However, factors in childhood such as chronic diseases, may have influenced educational attainment. Fourth, the selection of covariates is crucial, given the strong assumptions for mediation. The assumption requires that the proposed covariates be sufficient to control for confounding of the exposure-outcome, exposure-mediator, and mediator-outcome relationships [40]. We did not adjust for family history of FM, sleep problems and occupational factors [3] in our analyses, due to data unavailability. Thus, we cannot rule out residual confounding due to unmeasured or unknown confounders. We had missing data in our exposure variable and the mediators, for which we conducted multiple imputation, assuming that our data were missing at random. However, there is a possibility that some of our data may not have been missing at random, which could introduce bias in the results from the imputed datasets. We also cannot rule out missing on follow up that may have been due to symptoms related to FM, among those who had not yet been diagnosed during the study period.

## Implications & conclusion

The current study showed that lifestyle factors, particularly smoking and alcohol consumption, partly mediated the education gradient in self-reported FM. This result implies that women with different levels of education have differential exposures to these lifestyle factors. In addition, there may be some element of differential vulnerability across educational levels, given the alcohol-harm paradox observed with alcohol in our results. The study addresses a topic with paucity of research, and thus raises awareness about the relationship between education, lifestyle factors and FM. Knowledge of intermediate factors sheds light on possible mechanisms that may underlie educational disparity in self-reported FM. Also, interventions to adopt healthy lifestyle behaviors, targeted at women with low educational attainment, can contribute to reduce the educational disparity in FM and subsequently bridge the health gap. Future studies should investigate other dimensions of socioeconomic position, differential vulnerability, psychosocial, occupational, and other lifestyle factors such as dietary pattern. Also, application of a life course approach, can provide better understanding of the underlying mechanisms between socioeconomic position and fibromyalgia.

## Data Availability

The data that support the findings of this study are not publicly available. Ethical and legal restrictions apply to the availability of these data, which were used under license for the current study. However, researchers can apply for access to data from NOWAC study following guidelines provided at the website - https://uit.no/research/nowac. Enquiries about the NOWAC Study can be sent by email to the advisor - bente.isaksen@uit.no.

## Abbreviations

AGReMA: A Guideline for Reporting Mediation Analyses
BMI: Body Mass Index
CI: Confidence Interval
DE: Direct Effect
FM: Fibromyalgia syndrome
HR: Hazard Ratio
IE: Indirect Effect
NOWAC: Norwegian Women and Cancer
PA: Physical Activity
PY: Person-year
REK: Regional Committees for Medical and Health Research Ethics
SD: Standard Deviation
SEP: Socioeconomic Position
TE: Total Effect
